# Triceps Rupture and Repair in a Healthy, Young Woman following Rock Climbing

**DOI:** 10.1155/2021/3340479

**Published:** 2021-11-18

**Authors:** Charles Qin, Sean Clancy, Jason Strelzow

**Affiliations:** University of Chicago, Department of Orthopaedic Surgery and Rehabilitation, USA

## Abstract

Triceps tendon rupture in females is rare. In this case report, we present a young adult female patient with a distal triceps tendon rupture from bouldering treated with open surgical repair technique using a modified bone tunnel and suture anchor fixation technique. The diagnosis and technique for repair and postoperative rehabilitation are described. A review of the current literature of biomechanical and clinical outcomes of various repair techniques is also presented.

## 1. Introduction

Distal rupture of the triceps tendon is a rare injury. In the largest review of tendon ruptures, only 0.8% of 1,014 ruptures involved the triceps tendon [1]. The most common mechanism of injury found in the literature is a fall on an outstretched hand or during weightlifting, as contraction of the triceps against a flexed elbow eccentrically overloads the tendon leading to rupture [2, 3]. Risk factors for this injury include anabolic steroid use, local steroid injection, metabolic bone disease, and type 1 diabetes, among others [3–5].

Triceps tendon rupture in females is rare with a cited male predominance of 2 to 1 or 3 to 2 in all age groups [3]. Of note, most case series, although they should not be used to draw epidemiological conclusions, are written exclusively about male patients. A case series of 22 patients had only 3 females [6]. In another case series of 28 patients, 7 patients were female, all of whom were either elderly patients who sustained accidental falls or younger patients with high-energy injuries with associated injuries about the elbow such as radial head fractures and dislocations [7]. Moreover, bouldering, the activity that led to the injury in our case report, is a relatively niche activity and tendon injuries associated with this subdiscipline of climbing are infrequently reported.

The treatment of distal rupture of triceps tendon is an evolving target. In a recent review article, nonsurgical management was recommended for partial tears, tears at the musculotendinous junction, and tears with insignificant loss of extension strength [8, 9]. Conversely, surgical management is recommended for acute complete tears at the tendinous insertion with significant loss of extension strength.

In this case report, we present a young adult female patient with a distal triceps tendon rupture from bouldering treated with open surgical repair technique using a modified bone tunnel and suture anchor fixation technique.

## 2. Case Report

**Figure 1:**
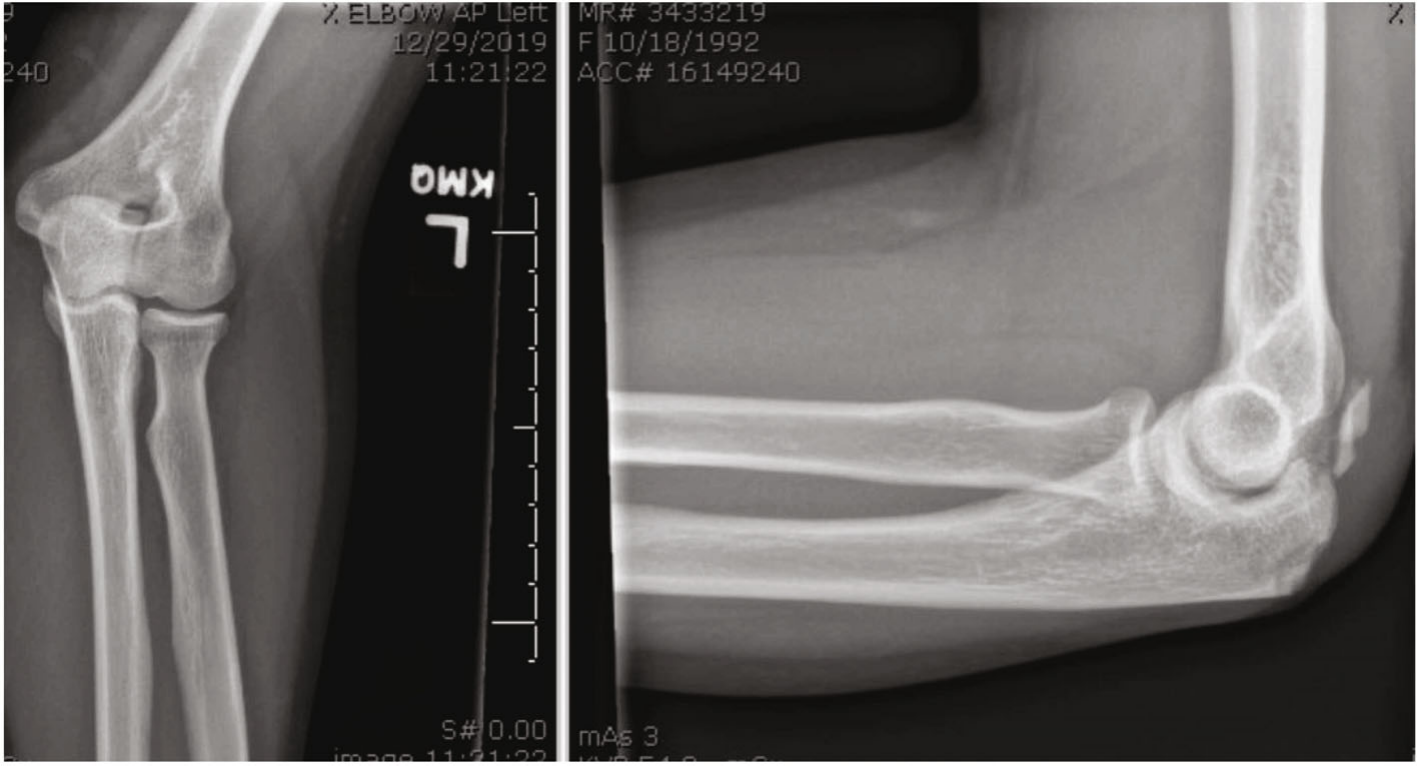


A 27-year-old otherwise healthy right hand dominant female patient presented to clinic 9 days after falling while bouldering with a left elbow injury. Initial management included splinting at a local emergency department before presentation to our clinic. She denied use of anabolic steroids or prior injections to her triceps tendon. On physical exam, she had ecchymoses over her olecranon, a palpable gap, mobile bony fragment, and inability to actively extend her elbow. Left elbow x-rays demonstrated an avulsion fracture of the proximal olecranon with moderate soft tissue edema (Figure 1). After a patient centered discussion, surgical repair was recommended as the patient was very eager to return to her preinjury functional activity level.

**Figure 2:**
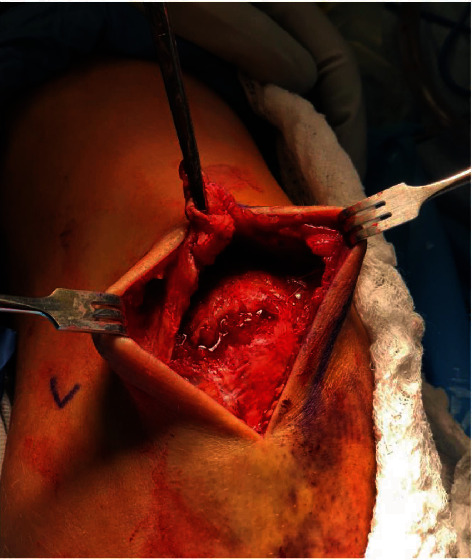


**Figure 3:**
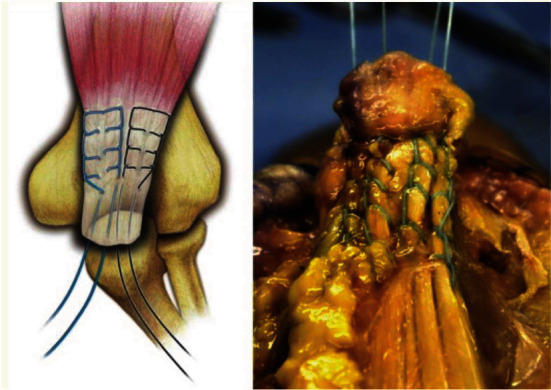


The patient was administered regional anesthesia and MAC, positioned laterally with the arm over a bolster. A tourniquet was used throughout the case. A midline posterior incision to the triceps was performed down to fascia exposing an obvious defect in the triceps tendon, a bony avulsion fracture attached to the triceps tendon, and a bald proximal olecranon corresponding to the tendon insertion. Inflammatory tissue of the tendon and avulsion fracture bed was debrided. The exposed tendon and fracture bed are shown in Figure 2. The triceps was whip-stitched with locking suture (No. 5 FiberTape Arthrex Naples, Florida), and 2 parallel 2.0 mm bone tunnels were drilled 2.5 cm from the tip of the olecranon as shown in Figure 3 and described by Paci et al. [10] The tails of the whip stitch suture were pulled through these bone tunnels using the Heuston suture passer and secured to a 4.5 mm Swivelock suture anchor (Arthrex Naples, Florida) that was drilled distally in the proximal olecranon as a knot-less anchor point. Once satisfactory tension of the sutures was obtained with the elbow in near full extension, the elbow was ranged to remove creep from the system. The suture repair was then secured with the anchor inserted into bone. The triceps fascia was closed with Maxon suture, followed by interrupted deep dermal and finally running subcuticular closure.

**Figure 4:**
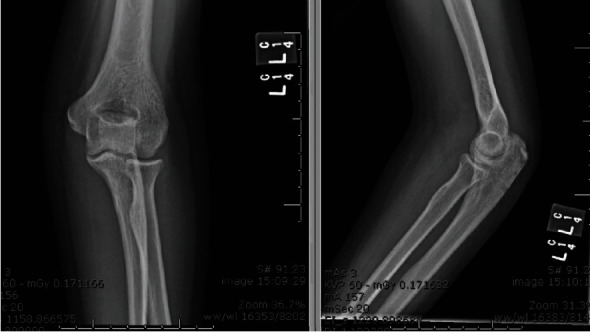


Postoperatively, the elbow was immobilized in 20 degrees of flexion in a plaster splint to detension the repair. Instructions were given for elevation and immediate range of motion (ROM) of the fingers, wrist, and pendulum of the shoulder. Postoperative rehabilitation started at 2 weeks. No resistance or passive ROM was employed. X-rays obtained 2 weeks postoperatively demonstrated the avulsion fragment in anatomic position on the olecranon with early callus present (Figure 4).

A custom fabricated orthotic restricting flexion to 30 degrees was fabricated for the patient on the day of surgery. The allowable range of motion was advanced by 10 degrees of flexion per week, and the orthotic was discontinued at 6 weeks. Due to the global pandemic, strengthening was progressed via virtual telemedicine visits.

**Figure 5:**
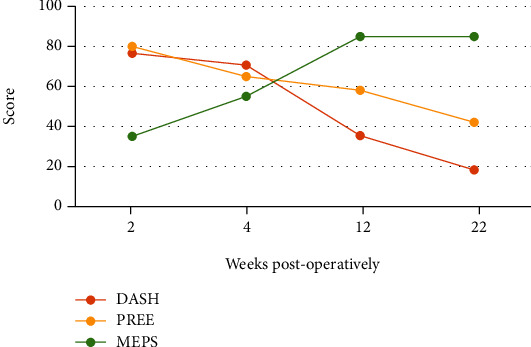


The Disabilities of Arm, Shoulder and Hand (DASH) score, Patient-rated Elbow Evaluation (PREE) score, and Mayo Elbow Performance Score (MEPS) and active ROM were collected at 12 and 22 weeks postoperatively (Figure 5) [11–13]. At 12 weeks, the DASH score was 35.3, PREE score was 58, MEPS was 85, and active ROM was 5-140. At 22 weeks, the DASH score was 18.3, PREE score was 42, MEPS was 85, and active ROM was 3-140. She has now returned to advanced yoga and strengthening exercises without limitation to her elbow and triceps.

## 3. Discussion

Triceps tendon ruptures are exceedingly rare [1]. While a 2 to 1 ratio of males to females is cited, distal rupture of the triceps tendon in females may occur much less frequently [3]. Mirzayan et al. presented a multicenter retrospective review of 181 patients with distal triceps ruptures with a mean age of 49, of whom 12 were female [14]. The most common mechanisms of injury were fall and weightlifting. Waterman et al. reviewed the outcomes of 88 patients undergoing distal triceps tendon repair with a mean age of 48, of whom only 5 were females [15]. In the only series that specifically reported both the age and injury mechanism of each of their female patients, 5 were elderly individuals who sustained a ground level fall and only 2 females were under the age of 60 [7]. Based on the limited available literature on this topic, it would appear that traumatic triceps tendon ruptures in young females without concomitant elbow injuries are extremely uncommon. Nonetheless, basic science data supports a greater susceptibility to tendon injuries in females compared to males (Magnussen et al.). Estrogen levels have been shown to affect the acute exercise-related synthesis of collagen. Moreover, the tendon hypertrophy response to loading has been observed to be attenuated in females.

The traditional surgical technique of triceps tendon rupture repair is a transosseous cruciate construct where locking sutures are passed through crossed bone tunnels and tied over a bone bridge. van Riet et al. published the outcomes of 14 primary repairs performed with a transosseous technique, 3 of which were noted to have rerupture (21%) [6]. Recent advances have led to the development of suture anchor repair techniques. Potential pitfalls of suture anchor fixation include violating the ulnohumeral joint as well as bursal and cutaneous irritation from the suture anchors and subcutaneous knots in the absence of knotless fixation. In an attempt to minimize these pitfalls while taking advantage of the biomechanical properties of suture anchors, we performed a repair combining 2 bone tunnels and 1 knotless suture anchor for an anatomic distal triceps repair as described by Paci et al. [10]

Biomechanical outcomes following transosseous and suture anchor repair of the triceps tendon are mixed. Studies have demonstrated the superiority of suture anchor fixation with a double row technique [16]. Specific to the repair technique used in this case report, Clark et al. found that the repair was more resistant to displacement compared to the traditional transosseous repair [17]. However, the differences seen in biomechanical properties may be affected by the unequal number of sutures across the repair site for different constructs. When Carpenter et al. compared transosseous cruciate with knotless suture anchor repair with equal number of sutures, no differences in displacement were found [18]. Nonetheless, the literature does support the technique used in this case as a viable construct for surgical treatment of triceps tendon ruptures.

A retrospective study of 56 patients undergoing triceps tendon repair with minimum 2-year follow-up reported no minimal clinically important difference in functional outcomes or rerupture rate between patients undergoing transosseous repair and suture anchor repair [19]. In a larger series of 181 patients, functional outcomes were not recorded but the authors did report significantly greater rerupture rate (6.7% vs. 0.0%) in patients undergoing transosseous repair in comparison to suture anchor repair. Given that there is a low incidence of distal triceps injuries and no single described repair technique has been shown to be superior in terms of biomechanical properties and clinical outcomes, it is unsurprising that the above-mentioned studies are not standardized with regard to the type and number of sutures, number of suture anchors used, or rehabilitation protocols. Specific to rehabilitation protocols, the literature espouses immobilization from anywhere between 2 and 6 weeks depending on individual surgeon assessment of intraoperative suture tension with passive exercises to follow [7, 15]. Future investigation attempting to standardize these factors is warranted.

We have presented a 27-year-old healthy female patient who sustained an avulsion distal triceps tendon injury which was repaired with knotless suture anchor fixation.

## Data Availability

The authors can make data available upon request.
